# The New Frontier of Host-Directed Therapies for *Mycobacterium avium* Complex

**DOI:** 10.3389/fimmu.2020.623119

**Published:** 2021-01-22

**Authors:** Nathan P. Crilly, Samuel K. Ayeh, Petros C. Karakousis

**Affiliations:** ^1^Department of Molecular and Comparative Pathobiology, Johns Hopkins School of Medicine, Baltimore, MD, United States; ^2^Department of Medicine, Johns Hopkins University School of Medicine, Baltimore, MD, United States; ^3^Department of International Health, Johns Hopkins Bloomberg School of Public Health, Baltimore, MD, United States

**Keywords:** nontuberculous mycobacteria (NTM), *Mycobacterium avium* complex, host-directed therapy, *Mycobacterium tuberculosis*, drug repurposing

## Abstract

*Mycobacterium avium* complex (MAC) is an increasingly important cause of morbidity and mortality, and is responsible for pulmonary infection in patients with underlying lung disease and disseminated disease in patients with AIDS. MAC has evolved various virulence strategies to subvert immune responses and persist in the infected host. Current treatment for MAC is challenging, requiring a combination of multiple antibiotics given over a long time period (for at least 12 months after negative sputum culture conversion). Moreover, even after eradication of infection, many patients are left with residual lung dysfunction. In order to address similar challenges facing the management of patients with tuberculosis, recent attention has focused on the development of novel adjunctive, host-directed therapies (HDTs), with the goal of accelerating the clearance of mycobacteria by immune defenses and reducing or reversing mycobacterial-induced lung damage. In this review, we will summarize the evidence supporting specific adjunctive, HDTs for MAC, with a focus on the repurposing of existing immune-modulatory agents targeting a variety of different cellular pathways. We also highlight areas meriting further investigation.

## Introduction

Nontuberculous mycobacteria (NTM), including organisms of the *Mycobacterium avium* complex (MAC), represent a significant and growing threat to human health worldwide. Since the beginning of the AIDS epidemic in the 1980s, the prevalence of MAC infection has increased substantially worldwide (1). MAC is widely distributed in the environment, including in water and soil, and is transmitted via inhalation into the respiratory tract and via ingestion into the GI tract (2). The most common clinical syndromes caused by MAC are pulmonary infection in patients with underlying lung disease, as well as disseminated disease in the severely immunocompromised (3, 4). A recent review of MAC pulmonary disease worldwide reported a five-year all-cause mortality rate of 27% (5).

In addition to the virulence factors common to all mycobacteria, MAC possesses several unique features which may contribute to pathogenesis. For example, MAC demonstrates increased resistance to phagosome-lysosome fusion and oxidative damage in murine macrophages, suggesting a unique ability to survive within activated macrophages (6). MAC can escape from macrophages undergoing apoptosis and survive extracellularly, evading the cytotoxic response necessary to eliminate intracellular bacteria (7). MAC also expresses several unique glycopeptolipids, which may modulate macrophage signaling cascades, thereby preventing an effective inflammatory response (8).

Treatment of MAC is challenging. Current treatment recommendations vary depending on the underlying conditions, severity of disease, and *in vitro* susceptibility profile. Macrolide-susceptible pulmonary disease is generally treated with a three-drug regimen, which includes a macrolide, ethambutol and a rifamycin, for at least 12 months after negative sputum-culture conversion (9). MAC often exhibits resistance to first-line antibiotics, and *in vitro* susceptibility testing for non-macrolide drugs has poor correlation with clinical efficacy. MAC pulmonary infection can present as cavitary disease with long-term respiratory sequelae. A milder form of the disease, which manifests as fibronodular bronchiectasis has a slower progression, but has been linked to increased mortality (10).

In the face of the increasing prevalence, high mortality, and treatment challenges associated with MAC infections, new therapeutic options are urgently needed. A promising avenue of research is that of host-directed therapies (HDTs). HDTs are adjuncts to antimicrobial therapy, differing from the latter in that they target host processes rather than the pathogen itself. The goal of HDTs is to boost protective immune responses, especially those inhibited or otherwise modified by the pathogen, and prevent excessive pathological inflammation (11, 12). Unlike novel antibacterial agents, they also confer the advantage of not contributing to drug resistance or cross-resistance to conventional antibiotics (12). Although HDTs are an active area of investigation in the therapy of tuberculosis (TB), as well as many non-mycobacterial infectious diseases (11–15), there has been a relative dearth of research into the potential of HDTs as adjunctive therapies for disease caused by MAC (16).

In the current review, we summarize HDT agents which are currently under investigation for MAC disease, as well as other HDTs and potentially targetable host pathways, which have not been investigated directly for MAC, but which show promise for future research.

## Improvement of Antimycobacterial Immunity

### Enhancing Autophagy: mTOR Inhibitors

Autophagy is a key self-degradative process in which the cytoplasmic contents of a cell are taken up by autophagosomes, trafficked to the lysosome, and digested (17). Although basal levels of this process occur in every cell, stress conditions, such as nutrient deficiency or pathogen infection, induce autophagy as a way of establishing homeostasis (18, 19). Autophagy plays a role in multiple physiological and pathological pathways, including the clearance of mycobacteria and other intracellular pathogens (17).

Initiation of autophagy is dependent on the Unc-51-like kinase-1 (ULK1) complex. This initiator complex is, in turn, regulated by the master regulator of autophagy, mammalian target of rapamycin (mTOR). mTOR plays a critical role in cellular metabolism, promoting anabolism and suppressing catabolic processes, such as autophagy (20). mTOR signaling is complex and can be activated or inhibited by a wide variety of molecules and signaling pathways. Nutrient states, particularly amino acid levels at the cellular level, serve as the main signal for mTOR activation. In nutrient-rich states, mTOR exerts an inhibitory effect on the ULK1 complex, leading to suppression of autophagy (21). Because of its important role in metabolism and cell growth, mTOR inhibition is a therapeutic target for a number of diseases, including autoimmune disorders and cancer (22). Rapamycin and other analogs directly inhibit mTOR activity, and vitamin D blocks upstream signaling to activate mTOR (22, 23). During *Mycobacterium tuberculosis* (Mtb) infection, the activation of both intracellular or extracellular surface pattern recognition receptors (PRRs) by certain unique Mtb-associated molecules, such as lipomannan, lipoarbinomannan, phthiocerol dimycocerosate (PDIM), lipoproteins, mycolic acid and *Mtb* DNA/RNA, induces autophagy (24, 25). Given that autophagy plays an important role in mycobacterial clearance, and MAC can survive intracellularly by blocking phagosome-lysosome fusion, enhancing autophagy through inhibition of the mTOR pathway appears to be an attractive HDT strategy (26, 27).

To date, there has been little research on targeting autophagy to improve host control of MAC infection. Early *et al*. reported that induction of autophagy by lactoferrin increases MAC killing by macrophages and renders the bacteria more susceptible to ethambutol, suggesting that autophagy is worthy of further investigation as an HDT target (28). Although they have not been studied in the context of MAC infection, mTOR inhibitors have been explored as HDTs for Mtb, with mixed results (29). Most data from *in vitro* studies have suggested that mTOR inhibition may result in enhanced intracellular killing of Mtb, however there is also some contrasting evidence to suggest that induction of autophagy results in increased Mtb growth, especially in the context of Mtb/HIV co-infection (30, 31). Vitamin D, an upstream inhibitor of mTOR signaling, also has shown some promise as an HDT for TB, although clinical trials do not show a consistent benefit, and it has not been investigated specifically against MAC (32).

Aside from autophagy, mTOR is involved in multiple metabolic and immunological pathways, which could affect mycobacterial pathogenesis and immunity. As a whole, the role of mTOR and autophagy in MAC infection remains largely unexplored, and further research is required to evaluate its suitability as an HDT target.

### Blocking the PD-1/PD-L1 Pathway: Anti-PD-1/PD-L1 Therapy

The Programmed Cell Death Protein-1 (PD-1) and its ligand, PD-L1, are the major components of the PD-1/PD-L1 pathway, an immune checkpoint, which regulates peripheral immune tolerance and suppresses inflammation (33). PD-1 is expressed on multiple cell types, including activated T cells, B cells, natural killer cells, and macrophages. PD-L1 is expressed on nonlymphoid cells. Binding of PD-1 to PD-L1 inhibits proliferation and effector functions of T and B cells, preventing self-reactivity (34). PD-L1 is highly expressed on tumor cells and virus-infected cells, conferring resistance to cell-mediated immunity. PD-L1 is also expressed on macrophages and plays a role in regulating immunosuppressive and pro-inflammatory activity. PD-L1 signaling in tumor-associated macrophages induces an immunosuppressive phenotype (35). Recently, the PD-1/PD-L1 pathway has become the subject of extensive research in cancer immunotherapy, as PD-1/PD-L1 antibody blockade has demonstrated efficacy in inducing cell-mediated immunity against multiple cancer types. Treatment of tumor-associated macrophages with anti-PD-L1 antibodies confers a pro-inflammatory phenotype, with increased expression of inducible nitric oxide synthase (iNOS), MHC II, TNF-α, and CD40 (36, 37). This is particularly important, since TNF-α and iNOS are critical effector mechanisms in the killing of intracellular mycobacteria, including MAC by macrophages (38). In patients with MAC pulmonary disease, expression of PD-1 by CD4 T cells is directly correlated with disease severity (39). An analysis of peripheral blood mononuclear cell (PBMC) function in such patients found that expression of PD-1 and PD-L1 were increased in lymphocytes of infected patients, which correlated with increased lymphocyte apoptosis compared to lymphocytes from healthy controls (40). Treatment of PBMCs obtained from MAC patients with anti-PD-1 and PD-L1 antibodies resulted in increased IFN-γ production and reduced T-cell apoptosis compared to PBMCs from healthy controls (40). These data suggest that PD-1/PDL-1 therapy could rescue immune cells from an immunosuppressive phenotype, allowing an improved immune response against MAC.

Although anti-PD-1 therapy may hold promise for treatment of MAC, there is some evidence that PD-1 is necessary for mycobacterial immunity, particularly against Mtb. Thus, mice deficient in PD-1 are more susceptible to Mtb infection (41). In Mtb granulomas, PD-1 is expressed in stable, cellular granulomas, but not in caseating ones, suggesting that it plays a role in granuloma maintenance. In a three-dimensional cell culture model, PD-1 inhibition led to increased Mtb growth, possibly due to excessive TNF-α expression (42).

The potential of anti-PD-1/PDL-1 therapy to improve the immune response to MAC remains to be investigated, both *in vitro* and *in vivo*. As anti-PD-1/PD-L1 therapy becomes more common in cancer therapy, retrospective analyses of its effect on patient susceptibility to MAC disease and clinical outcomes following MAC therapy may be useful.

### Heme Oxygenase Inhibition

Heme oxygenase (HO-1) is an antioxidant enzyme that catalyzes the conversion of heme into carbon monoxide, biliverdin and iron (43, 44). Apart from its role in cytoprotection, HO-1 has been shown to regulate cell proliferation, differentiation, and apoptosis (44). The induction of pulmonary HO-1 is associated with TB disease (45), suggesting its potential utility as a diagnostic biomarker. Although its role in TB pathogenesis is not fully understood, experimental data in Mtb-infected mice have shown that lung bacterial loads decrease following HO-1 inhibition by the metalloporphyrin, SnPPIX (45). The same study found that a combination of an HO-1 inhibitor, SnPPIX and antimycobacterial therapy enhanced T-cell-dependent pathogen clearance. Clinical data have shown that plasma HO-1 levels decline following successful TB treatment (46).

As in the case of Mtb infection, HO-1 has been found to be elevated during MAC infection in BALB/c mice (47). Consistent with a host protective role in resisting MAC infection, mycobacterial burden in the liver, lungs and spleen was significantly higher and the disease was more likely to be disseminated in mice with HO-1 deficiency compared to HO-1 homozygous or heterozygous mice (47, 48). Further investigation is required to determine how HO-1 activity is regulated during MAC infection, and whether HO-1 inhibition is a promising HDT in the context of MAC.

### IFN-γ Therapy

IFN-γ plays a significant role in immunity against Mycobacterium infections. In contrast to type I IFNs (α and β), which are made by virus-infected cells, IFN-γ is produced by activated T cells, NK cells, and macrophages, leading to the activation of phagocytes, stimulation of antigen presentation to T cells, and regulation of several other cellular functions, including proliferation, apoptosis, and cell adhesion (49). In particular, IFN-γ induces the expression of iNOS (50) and the respiratory burst enzyme NADPH-dependent phagocyte oxidase (51), thereby enhancing the mycobactericidal activity of macrophages. Mice with mutations in the IFN-γ receptor have been shown to have increased susceptibility to intracellular pathogens (52). Pre-treatment of intestinal and peritoneal-derived macrophages with IFN-γ produced both bactericidal and bacteriostatic activity against MAC following infection of these cells (53, 54). Although *in vivo* treatment of beige and Swiss-Webster mice with recombinant murine IFN-γ did not alter the course of visceral MAC infection (55), the bactericidal activity of clofazimine against MAC was enhanced in beige mice pre-treated with IFN-γ (54).

Mutations in the IFN-γ receptor gene or anti-IFN-γ autoantibodies confer increased susceptibility to disseminated NTM infections in humans (56–58). IFN-α, which, like IFN-γ, signals through STAT1, activating many common downstream effector genes, has shown some promise in treating patients with IFN-γ signaling defects and disseminated mycobacterial disease (59). In a study of 7 patients with disseminated MAC infection, subcutaneous administration of IFN-γ, in combination with conventional medical treatment, resulted in improvement in symptoms, and pathological and radiological findings, and also reduced the need for medical procedures, such as paracentesis following 8 weeks of treatment (60). Aerosolized IFN-γ has shown some promise in treating patients with TB and idiopathic pulmonary fibrosis, and is worthy of study in patients with pulmonary MAC (61).

## Prevention of Excessive and Pathological Inflammation

### Suppressing Excessive TNF-α Activation: Anti-TNF Antibodies

Tumor necrosis factor alpha (TNF-α) is a pro-inflammatory cytokine which is upregulated during MAC and Mtb infection and plays an essential role in antimycobacterial immunity (62). During mycobacterial infection, T cells, macrophages, and dendritic cells produce TNF-α in response to multiple signaling pathways (63). TNF-α signaling is complex, and the cytokine serves multiple functions, including in the formation and maintenance of granulomas, as evidenced by the observation that mice deficient in TNF-α or receiving anti-TNF-α therapy produce defective granulomas following mycobacterial infection (64, 65). TNF-α also promotes killing of intracellular mycobacteria by macrophages, as the TNF blockers adalimumab and infliximab suppressed phagosome maturation in primary human PBMCs in the presence or absence of IFN-γ (66) Moreover, TNF-α serves macrophage antimicrobial functions by activating reactive oxygen and nitrogen species (67). Treatment with anti-TNF-α antibody has been associated with decreased resistance to MAC infection in mice (68).

Although TNF-α is required for an effective immune response, excessive TNF-α production has deleterious pathological effects. Thus, when its production is properly regulated, TNF-α induces apoptosis of Mtb-infected infected cells by recruiting Fas-associated protein with death domain (FADD) and subsequent activation of effector caspases and signal-regulating kinase 1 (ASK1), thus favoring mycobacterial clearance (63, 69, 70). However, when produced in excessive amounts, TNF-α results in necrosis of Mtb-infected macrophages and hyperinflammation through activation of serine/threonine-protein (RIP)1/3 kinases and mitochondrial reactive oxygen species (ROS) production (70–72). TNF-α also induces necroptosis, a highly inflammatory form of cell death, which could contribute to pathological inflammation (73).

Because of its roles in mycobacterial immunity and pathology, TNF-α has been a focus of HDT investigation. Multiple anti-TNF antibodies and TNF soluble receptors have been approved for use in humans to block TNF-α activity, and are primarily used to treat autoinflammatory conditions, such as rheumatoid arthritis. TNF blockers have shown some promise as HDTs for mycobacterial infections. Combined use of the TNF-α receptor inhibitor etanercept with antibiotics decreased the lung burden of Mtb and reduced TB-associated lung pathology in infected mice compared to antibiotics alone (74). However, the role of anti-TNF therapy in clinical cases of mycobacterial infection is controversial. Patients receiving anti-TNF therapy are at increased risk for developing disease due to Mtb and MAC (75–77). After a diagnosis of TB or MAC disease is made, anti-TNF therapy is usually halted at least until anti-mycobacterial therapy has been initiated and the infection is under control. On the other hand, there are several reports of TB patients experiencing clinical exacerbation upon discontinuation of anti-TNF treatment, and improvement of disease following its reinstitution (78–80). In addition, a subset of MAC-infected patients show favorable outcomes if anti-TNF therapy is maintained throughout treatment (76). However, it is uncertain in these cases whether anti-TNF therapy contributed as an adjunctive HDT or by ameliorating the underlying autoimmune disease.

The roles of TNF-α in mycobacterial immunity and disease are complex, and the therapeutic potential and risk of inhibiting TNF-α function during MAC infection require further investigation. Given the relatively long half-lives of most TNF blockers relative to antibiotics, there is concern over sudden stoppage of all treatment by patients, resulting in the unopposed anti-TNF activity and possible worsening of infection (81). Since TNF-α interacts with multiple other signaling pathways, further research is also needed to identify other cytokines which, if targeted in tandem with TNF-α, could hold promise as HDTs.

### Broad Suppression of Inflammation: Nonsteroidal Anti-Inflammatory Drugs and Corticosteroids

Excessive and chronic inflammation is an important factor in the progression of mycobacterial disease (82). Thus, the broad inhibition of the inflammatory response by non-steroidal anti-inflammatory drugs (NSAIDs) or corticosteroids is an attractive HDT strategy. NSAIDs have been well-studied as adjunctive therapies for TB, with a protective effect, both in animal models and in human disease, when used in conjunction with antibiotics (83). There are multiple proposed mechanisms for these effects. NSAIDs suppress the excessive recruitment of neutrophils to granulomas, which can be responsible for destructive inflammation (84, 85). By reducing prostaglandin E2 (PGE2) expression, NSAIDs also inhibit phagocytosis and killing of mycobacteria during late TB (86). NSAIDs have anti-thrombotic effects, which may prevent the hypercoagulable state occasionally observed with severe TB (87, 88). Despite their relatively well-characterized role as an adjunctive therapy for TB, there has been little research into NSAIDs as HDTs for MAC. The NSAID diclofenac sodium modulates multiple cytokines in MAC-stimulated macrophages but does not improve bacterial clearance by macrophages or infected mice (89). Although NSAIDs can prevent destructive inflammation, they might also inhibit an effective immune response. This is especially concerning for MAC, since an immunocompromised state is a major risk factor for disseminated MAC disease (10). NSAIDs have not been causally linked to MAC disease, but long-term NSAID use has been identified as a possible predisposing factor in at least one case (90).

Corticosteroids are some of the earliest HDTs used for mycobacterial disease and may be useful in treating patients with late-stage and extrapulmonary TB (91, 92). In particular, short-term steroid use, by reducing inflammation caused by antibiotic-mediated killing of mycobacteria and accompanying increased intracranial pressure, has been shown to improve mortality by as much as 25% in patients with tuberculous meningitis (93). Similar to NSAIDs, the beneficial effect of corticosteroids is primarily attributed to the suppression of pathological inflammation. Corticosteroids exert their anti-inflammatory effects through a variety of mechanisms, including by inducing transcription of anti-inflammatory genes, such as annexin-1, IL-10 and IκB-α (inhibitor of NF-κB), by direct interacting with NF-κB, AP-1 and other immunomodulatory transcription factors, inhibiting maturation and differentiation of antigen presentation cells with reduced sensitivity to T cell regulation, and promoting the formation of macrophages with anti-inflammatory properties (94).

The use of corticosteroids as an HDT for MAC disease is somewhat controversial, due to their immunosuppressive effects and the lack of controlled studies (95–97). Although there is a significant body of research on the use of corticosteroids in reducing inflammation due to a variety of infectious diseases, their specific role as an adjunctive HDT for MAC disease has not been studied. Further research is required to understand the effects of corticosteroids on MAC infection on the molecular, cellular, and organismal level, to determine whether their use is justified or contraindicated in specific stages of MAC disease.

## Multiple Mechanisms of Action

### Targeting Lipid Metabolism and Inducing Autophagy: Statins

The 3-hydroxy-3-methylglutaryl coenzyme A (HMG-CoA) reductase inhibitors (statins) are a class of lipid-lowering medications, which have shown promise as HDTs for TB (98). PBMCs from patients with familial hypercholesterolemia receiving statin therapy demonstrate resistance to *ex vivo* Mtb infection compared to those from untreated donors (99). Adjunctive therapy with simvastatin enhanced the bactericidal activity of the first-line anti-mycobacterial regimen in a mouse model of chronic TB and shortened the duration of curative treatment in a murine model of TB relapse (100, 101). Consistent with a class effect of statins, pravastatin adjunctive therapy showed a dose-dependent reduction in bacillary lung burden and decreased lung inflammation in conjunction with front-line chemotherapy in a mouse model of chronic TB (102). Mechanistically, statins reduce the formation of lipid droplets in foamy macrophages, which may serve as a nutrient source for intracellular Mtb and contribute to antibiotic tolerance (99, 103). However, the primary HDT mechanism of action of statins likely involves the promotion of phagosome maturation and autophagy, thereby improving clearance of Mtb by infected macrophages (99). Statins enhance autophagy of Mtb-infected macrophages by blocking mTORC1, activating AMP-activated protein kinase (AMPK) and favoring nuclear translocation of transcription factor EB (TFEB) (104). Although the role of lipid-laden, foamy macrophages in MAC pathogenesis is less well understood than in TB, morphologically similar phenotypes have also been described in MAC-infected macrophages, and it is possible that statins could have similar HDT effects (105).

### Activation of AMPK and Potentiation of Macrophage Effector Function: Metformin

Multiple studies have found that use of the anti-hyperglycemic drug metformin reduces the risk of TB and improves clinical outcomes in patients with diabetes mellitus (106, 107). Experimental evidence indicates that metformin has multiple host-directed effects, which may promote clearance of MAC. The drug enhances mycobacterial killing in human PBMCs by promoting autophagy and phagosome-lysosome fusion, as well as by selectively increasing mitochondrial ROS production (108). Metformin has a dose-dependent inhibitory effect on intracellular replication of mycobacteria through activation of the adenosine monophosphate-activated protein kinase (AMPK) signaling pathway (109). Metformin also suppresses TNF-α expression in human monocytes (110). In Mtb-infected mice, metformin adjunctive therapy is associated with reduced chronic lung inflammation, enhanced immune responses, and improved efficacy of antibiotics (111, 112). In contrast, Dutta *et al*. showed that adjunctive therapy with human-equivalent doses of metformin did not enhance the bactericidal or sterilizing activities of the first line antitubercular regimen in Mtb-infected BALB/c mice (111). Given the widespread use of metformin and the high prevalence of MAC disease, retrospective analyses of the effect of metformin on MAC microbiological and clinical outcomes would be useful to gauge its promise as an adjunctive HDT for MAC.

### Immunomodulation and Antimicrobial Properties: Clavanin-MO

Clavanin-MO is a naturally occurring antimicrobial peptide which possesses immunomodulatory properties (113). Both *in vitro* and *in vivo*, clavanin-MO stimulates production of inflammatory mediators, including IFN-γ, granulocyte-macrophage-stimulating factor, and monocyte chemoattractant protein-1, while suppressing the pro-inflammatory cytokines IL-12 and TNF-α (113). Clavanin-MO protects animal models from infection by both gram-positive and gram-negative bacteria (113). Although clavanin-MO has not been tested against mycobacteria, its immunomodulatory effects could potentially improve the immune response against MAC while blocking pathological inflammation, especially since it affects both IFN-γ and TNF-α, which are targets of other promising HDTs.

### Potentiation of Macrophage Effector Function and Antimicrobial Activity: Thioridazine

Thioridazine is a neuroleptic drug, which has both direct antimycobacterial and host-directed effects (114, 115). The drug acts directly against Mtb by inhibiting antibiotic efflux pumps, thereby enhancing antibiotic susceptibility *in vitro* (116). Thioridazine also affects the host by inhibiting mammalian efflux pumps in the macrophage, leading to acidification of the phagosome and improving mycobacterial clearance (114, 117). Although its efficacy as an adjunctive therapy in murine models of chronic TB is controversial (118, 119), thioridazine was found to reduce the emergence of isoniazid-resistant mutants in Mtb-infected mouse lungs following co-administration with the standard anti-TB regimen (120). Thioridazine has been suggested as an adjunctive therapy for MAC, but research in this area has been limited (121–123). A short course of thioridazine and moxifloxacin was sufficient to clear MAC from infected monocytes (122). However, the pharmacokinetics of thioridazine may prevent it from reaching effective concentrations in the lung, thus limiting its clinical utility in MAC pulmonary disease (121, 123).

## HDTs With Unknown or Poorly Understood Mechanisms of Action

### Poloxamer CRL-1072

Poloxamer CRL-1072 is a surfactant which makes mycobacteria more susceptible to some antibiotics, possibly through disruption of mycobacterial surface lipids (124). Its effects are especially pronounced in macrophages and mice compared to broth culture, suggesting that it has an effect on the host response to mycobacterial infection (124). The mechanisms of action of CRL-1072 are poorly understood. The surfactant induces production of nitric oxide in cultured human macrophages, leading to improved clearance of MAC (125). In addition, CRL-1072 induces production of IL-8 in human macrophages, a chemotactic factor which attracts neutrophils and T cells to the site of infection (126). To date, there has been little research on CRL-1072, and much remains unknown about its potential as an HDT. An important consideration is that, as a surfactant, CRL-1072 would likely have to be delivered topically to the lungs via inhalation. There is precedent for inhaled therapies for MAC with the recently FDA-approved Amikacin Liposome Inhalation Suspension (ALIS) (127).

### Picolinic Acid

Picolinic acid is a degradation product of L-tryptophan with metal-chelating properties (128). An oral formulation, chromium(III) picolinate is safe and available as a nutritional supplement (129–131). Experimentally, it has both antimicrobial and host-directed effects against MAC. Specifically, picolinic acid potentiates the antimicrobial effects of clarithromycin, rifampicin, and some fluoroquinolones against both extracellular and intracellular MAC, suggesting that it has direct antimicrobial activity, which may be due to its iron-chelating properties (132). When used together with IFN-γ, picolinic acid also triggers apoptosis of MAC-infected mouse macrophages, thereby inhibiting intracellular mycobacterial growth (133, 134). Picolinic acid may also increase expression of TNF-α and interleukin-1, improving macrophage effector function (135). On the other hand, picolinic acid does not upregulate production of β-defensin-1, free fatty acids, or reactive oxygen and nitrogen intermediates (136). Therefore, its potentiation of macrophage effector functions remains poorly understood.

## HDT Target Pathways for Future Investigation

### HIF-1α

Hypoxia-inducible factor-1 alpha (HIF-1α) is a key regulator of cellular metabolism in hypoxic environments and is involved in the immune response, even under normoxic conditions (137). HIF-1α is thought to play an important role in immunity to mycobacterial infection. In zebrafish, stabilization of HIF-1α protects against *M. marinum* infection (138). The protective effect is related to upregulation of IL-1β in macrophages, which results in increased nitric oxide production by neutrophils (139). There is also evidence that HIF-1α plays multiple roles in the macrophage response to Mtb infection by mediating IFN-γ-dependent genes, regulating immune effectors, shifting metabolism to aerobic glycolysis, and blocking excessive inflammation (140–142). In general, HIF-1α promotes a pro-inflammatory state, which may improve mycobacterial clearance early in infection, but also induces pathological inflammation and immune exhaustion during chronic infection.

HIF-1α has not been well-studied in the context of MAC infection. However, research on other mycobacteria suggests that HIF-1α is a double-edged sword. Whereas induction of HIF-1α promotes a pro-inflammatory state, which may improve mycobacterial clearance early during the course of infection, it can also lead to pathological inflammation and immune exhaustion during chronic infection (140, 143). Targeting the HIF-1α pathway (and its timing) as an HDT strategy for MAC remains to be investigated.

### Broadly Protective HDT Targets Against Intracellular Pathogens

A recent study screened FDA-approved drugs to identify HDT targets with broad protection against multiple intracellular pathogens (14). Three targets were identified which broadly protect THP-1 cells from intracellular bacteria: antagonizing G protein receptor (GPCR) signaling, interfering with intracellular calcium signaling, and disrupting membrane cholesterol distribution (14). Although mycobacteria have been shown to manipulate G-protein-coupled receptors to suppress epithelial signaling pathways (144) and to inhibit intracellular calcium signaling, leading to reduced phagosome-lysosome fusion and increased mycobaceterial survival within human macrophages (145), these cellular pathways have not been directly targeted by therapies, and represent an area of potential future investigation.

## Conclusions

Although HDTs represent a promising tool to improve MAC clinical outcomes, they have been the subject of little research to date. Looking to the future, there are several major challenges and opportunities in MAC HDT research which remain to be met. Two specific research needs are a better understanding of MAC pathophysiology to identify HDT targets, and improved model systems to allow investigation of potential HDTs.

An improved understanding of the host-pathogen interactions during MAC disease could reveal additional HDT targets. To date, the majority of HDTs against MAC fall into two general categories: improving immune effector function or modulating pathologic inflammation. The mechanism of several HDTs are not completely understood. A better mechanistic understanding of their function could improve our knowledge of MAC pathophysiology and identify new pathways to be targeted by HDTs. For example, the efficacy of statins in improving TB clinical outcomes suggests that the metabolism of mycobacterial-infected cells may be a promising area of investigation (102).

A lack of *in vitro* and *in vivo* experimental models of MAC infection has been a major barrier to research. Current model systems are not standardized, and do not always yield replicable or clinically useful results (146). Cell cultures cannot entirely recapitulate a disease which involves long-term, complex interactions between multiple cell types, tissues and organs, while murine models of NTM differ from human disease in their immune responses and granuloma structure, and generally do not sustain chronic infection unless immune suppression is induced (147). These deficiencies are especially important for investigating HDTs, which may target complex or human-specific pathways. Recent advances in model systems will inform future HDT research. *In silico* models could identify promising HDTs prior to the expense and difficulty of *in vitro* and *in vivo* experimentation. Recent developments in organoid models promise to allow better *in vitro* investigation of complex pathways involving interactions between multiple cell types and the extracellular matrix. For example, a three-dimensional granuloma model has recently been developed for Mtb and could be a valuable tool for investigating HDTs if adapted for MAC (148).

Finally, there is an unexplored need to investigate the use of HDTs in combination. To date, most studies have examined a particular HDT in isolation or in combination with antibiotics. Investigation of HDTs with potentially complementary mechanisms could identify therapeutic combinations that have a greater effect than the sum of their parts.

MAC is an emerging infectious disease of particular concern due to its rising prevalence, resistance to frontline antibiotics, and associated chronic morbidity and mortality (1, 5, 10). HDTs against MAC represent a promising but underexplored avenue of research, which could hold great potential in improving microbiological and clinical outcomes.

## Author Contributions

NC and PK conceived the work. NC, SA, and PK wrote the manuscript. All authors contributed to the article and approved the submitted version.

## Funding

This work was supported by NIH/NIAID grants UH3AI122309 and K24AI143447 to PK. The funders have no role in the content of this manuscript.

## Conflict of Interest

The authors declare that the research was conducted in the absence of any commercial or financial relationships that could be construed as a potential conflict of interest.
